# Invited papers

**DOI:** 10.1054/bjoc.2000.1348

**Published:** 2000-08-10


*British Journal of Cancer* (2000) **83**(Suppl 1), 3–9
© 2000 Cancer Research Campaign
doi: 10.1054/ bjoc.2000.1348, available online at http://www.idealibrary.com on IDEAL®

### S1 THERAPEUTIC VACCINES FOR LYMPHOMA Dr Ronald Levy, Stanford University School of Medicine, USA

The antigen binding receptor on the surface of a lymphocyte is composed of protein chains, each with constant and variable domains. A unique combination of variable regions is formed by genetic rearrangements which occur during lymphocyte differentiation and which mark the clonal progeny of each original mature B cell or T cell. Malignant lymphomas have antigen binding receptors which are unique to each tumor and are expressed uniformly by all members of the malignant clone. These receptors can be isolated from the tumors by hybridoma or by molecular cloning techniques. Monoclonal antibodies generated against the unique (idiotypic) portion of receptors from B cell tumors have been able to induce complete and durable remissions in patients with lymphoma. Alternatively, it is possible to use the receptors as an active vaccine to induce an immune response in the host against his own tumor. Animals bearing established tumors can be cured by a combination of chemotherapy and idiotype vaccination (J Immunol. 141:3227–3233, 1988). Moreover, patients with B cell lymphoma be induced to make immune responses against the idiotypes expressed by their tumors, and these immune responses have been associated with tumor regression (N Eng J Med 327:1209–1215). Clinical trials are under way in patients with Follicular Lymphoma in which idiotype vaccines custom made from each patient's tumor are prepared and administered after chemotherapy induced remission. The results of these ongoing trials will be presented and novel methods of vaccine production will be discussed.

### S2 NICE THE NATIONAL INSTITUTE FOR CLINICAL EXCELLENCE, M.D. Rawlins, 90 Long Acre, Covent Garden, London WC2E 9RZ

The National Institute for Clinical Excellence has been established to provide NHS health professionals with advice on delivering the highest standards of care to their patients. In delivering this, NICE will provide guidance in three areas:

- use of individual technologies after "appraisal"
- management of specific clinical conditions (clinical guidelines and referral protocols)
- clinical audit methods

**Appraisal of technologies** will indicate whether they are both clinically and cost effective; and whether, how, and when they should be used within the NHS. Guidance will be accompanied by simple methods of clinical audit.

**Clinical management programmes** will cover both approaches to individual conditions (clinical guidelines) and the circumstances where specialist advice should be sought (referral protocols). Both will also be accompanied by simple approaches to clinical audit.

**Clinical audit.** The Institute will further the development of clinical audit by proposing methodologies in the areas in which it has provided guidance; by acting as an information resource to NHS staff; by supporting 'core audit' with professional bodies; and by supporting national 'sentinel' audits.

- NICE's agenda is ambitious and its first priorities include:
- establishing the Institute
- starting its work programme
- gaining the confidence of its stakeholders

### S3 IS THERE A FUTURE FOR LUNG CANCER SCREENING? Nick Wald, St Bartholomew's Medical School, London EC1M 6BQ, UK

ABSTRACT NOT RECEIVED

### S4 ORGANISATIONAL AND TECHNICAL ADVANCES IMPROVE THE RESULTS IN LUNG CANCER: A SURGEON'S VIEW Francis Wells, Papworth Hospital, Papworth Everard, Cambridge CB3 8RE, UK

ABSTRACT NOT RECEIVED

### S5 THE IMPLICATIONS AND OPPORTUNITIES OF THE CHART TRIAL IN THE TREATMENT OF NON-SMALL CELL LUNG CANCER

MI Saunders*, Mount Vernon Hospital, Rickmansworth Road, Northwood, HA6 2RN, UK

**Purpose** To evaluate the implications of the randomised controlled trial of CHART versus conventional radiotherapy in the treatment of locally advanced non-small cell lung cancer.

**Method** A total of 563 patients were entered into the trial between April 1990 and April 1995. The patients were randomised to conventional radiotherapy 60 Gy in 6 weeks or CHART where they received 54 Gy in 36 fractions treating three times a day on consecutive days. The results of the study are considered with reference to survival, locoregional control, metastasis-free survival, acute and late morbidity, quality of life and socio-economic assessment.

**Results** Survival: There is a significant long term improvement in the survival for the CHART arm with a 2 year survival probability of 30% compared to 21% for the conventional arm for the whole group ($p = 0.008$) and 33% versus 22% in those with squamous cell carcinoma ($p = 0.0007$). This increase in survival was achieved by gaining a significant improvement in locoregional control and an increase in the metastasis-free survival. Two years metastasis-free survival for those with squamous cell carcinoma was 52% in CHART and 42% with conventional radiotherapy.

Dysphagia was the major acute morbidity and came on earlier with CHART, was more severe but settled satisfactorily in patients in both arms of the study. Frequency and severity of radiation pneumonitis was slightly less in the CHART arm. Considering late morbidity there was no incidence of radiation myelitis and at two years only 7% and 5% were considered to have symptoms of dysphagia related to radiotherapy in the CHART and conventional arm. There was no trend for pneumonitis to be more troublesome in CHART compared to conventional radiotherapy. There was no difference between the regimes in the short or long term when quality of life was considered.

Socio-economic factors were carefully considered and taking the data from all sources and looking at the difference between CHART and conventional radiotherapy the study showed that CHART was more expensive than conventional radiotherapy but the cost differential is only £697.79 at 1994 levels. A further analysis of the data by Soren Bentzen, as yet unpublished, has shown looking at the difference between the two curves there is again a life expectancy from CHART (truncated at 4 years) is 101 days. Cost per life year gained by the use of CHART to 4 years is £2,585. This makes CHART extremely cost-effective when compared to other interventions in medicine.

**Conclusion** CHART radiotherapy gives a true therapeutic benefit when compared to conventional radiotherapy. As the socio-economic study suggested it is cost effective particularly when compared to other interventions currently used in oncology.

### S6 DOES CHEMOTHERAPY IMPACT ON THE OUTLOOK OF NON-SMALL CELL LUNG CANCER?

Heine H Hansen, Dept of Oncology, Finsen Centre, National University Hospital, Rigshospitalet, Copenhagen, Denmark

While there is no established role for adjuvant therapy including chemotherapy in connection with surgery for stage I and II patients, the picture is different for patients presenting with stage III and stage IV non-small cell lung cancer. For stage III patients induction combination chemotherapy with platinum-containing regimen followed by radiotherapy has shown superiority to radiotherapy alone in both individual trials and meta-analyses. The latter demonstrated a 13% reduction in risk of death and an absolute benefit of 4% at 2 years for the combined modality treatment versus radiotherapy alone. Survival data from recent randomized trials have also indicated superiority for concomitant chemotherapy and radiotherapy rather than radiotherapy alone in stage III patients. With respect to neoadjuvant chemotherapy followed by surgery in selected stage III patients, 2 minor studies have demonstrated significant survival advantages for the combined modality approach.

Almost 50% of the patients with NSCLC present with advanced disease (stage IV) and for patients with good prognostic factors combination chemotherapy with 2 or 3 drugs results in improved survival and quality of life. In patients relapsing after initial treatment, often platinum containing chemotherapy, docetaxel has shown to improve survival and quality of life compared with best supportive care in one study.

Overall the results of non-surgical treatment of NSCLC remain highly unsatisfactory in spite of the introduction of a number of new agents in the early 1990s. New treatment approaches are needed and under investigation.

### S7 MOLECULAR GENETICS OF DRUG SENSITIVITY AND RESISTANCE IN A MOUSE LYMPHOMA MODEL

Clemens A Schmitt, Jordan Fridman and Scott W Lowe, Cold Spring Harbor Laboratory, Cold Spring Harbor, NY 11724, USA

To develop physiological *in vivo* models for studying tumor cell radio- and chemosensitivity, we are investigating the therapeutic response of spontaneous lymphomas occurring in the *Eμ-myc* transgenic mouse. These animals constitutively express *c-myc* in the B-cell lineage and typically succumb to B-cell lymphoma with associated leukemia. We hypothesized that the *Eμ-myc* transgenic would be a tractable model, since: (i) tumor burden can be easily monitored by lymph node palpation or blood smears; (ii) lymphomas are detectable long before the animal dies, (iii) large numbers of pure tumor cells can be isolated from mice undergoing therapy, (iv) therapy is performed in immunocompetent mice, and (v) lymphoma cells readily adapt to culture and can be transplanted into syngeneic mice. We have characterized the response of *Eμ-myc* lymphomas to several anticancer drugs, and have developed methods to facilitate the analysis of genetic factors involved in drug sensitivity and resistance. Specifically, we have examined the impact of *INK4a/ARF* mutations, *p53* mutations, and Bcl-2 overexpression on the treatment sensitivity of *Eμ-myc* lymphomas. These studies demonstrate that: (i) disruption of the *INK4a/ARF* locus produces aggressive, chemoresistant tumors by disabling p53, (ii) disruption of apoptosis during the course of tumor development can simultaneously produce drug-resistant tumors, (iii) the Bcl-2 oncoprotein produces a multi-drug resistant phenotype when assayed in short-term culture or *in vivo*, and (iv) the impact of Bcl-2 on drug resistance is reduced when assayed in culture-adapted cells and completely missed in the standard clonogenic survival assay. Our results highlight the importance of physiological test systems to study treatment sensitivity, and establishes a strategy for producing genetically-defined lymphomas to evaluate compounds directed against specific lesions. Of note, *INK4a/ARF* and *p53* mutations are associated with reduced treatment sensitivity in human hematologic malignancies, implying the information obtained from our model will be applicable to human cancers.

### S8 CONVERGING ONCOGENES IN CANCER

Gerard Evan, University of California, San Francisco, Comprehensive Cancer Center, 2340 Sutter Street, San Francisco, California 94143-0128.

Cancers arise through accumulation of mutations that compromise control of cell proliferation, differentiation, cell adhesion and apoptosis. Deregulation of the cmyc proto-oncogene is a ubiquitous neoplastic mutation that disrupts cell growth control and renders cells independent of mitogens for cell cycle progression. However, activation of c-myc also sensitises cells to apoptosis. We believe that this innately contradictory action of c-myc acts as a restraint to the propagation of neoplastic cells within the soma, but this has never been directly demonstrated in vivo. We have therefore constructed mice harbouring a switchable Myc protein in specific tissues – specifically, T lymphocytes, pancreatic islets cells and suprabasal keratinocytes – to permit analysis of the immediate as well as the delayed consequences of c-Myc activation in a normal somatic cell. In pancreatic ß cells, c-Myc activation induces ~100% entry of cells into cell cycle. However, this is accompanied by massive apoptosis that overwhelms proliferation leading to islet involution and acute diabetes. Thus, neoplasias cannot arise in ß cells without early suppression of apoptosis. Indeed, co-expression of Bcl-2 blocks the apoptosis and, together with c-Myc, rapidly leads to dramatic ß cell hyperplasia. By contrast, activation of c-Myc in suprabasal keratinocytes triggers cell proliferation and blockade of keratinocyte differentiation with no ‘compensatory’ apoptosis. The result is a complex neoplastic papilloma phenotype exhibiting hyperplasia, dysplasia, neo-angiogenesis and parakeratosis. The absence of apoptosis in these papillomas is not because c-Myc lacks the facility to trigger keratinocyte cell death. Rather, it appears to be because of the presence of excess survival signals in intact epidermis: c-Myc is highly effective at inducing apoptosis in transgenic keratinocytes explanted into cell cultures devoid of survival factors. The strong implication from this is that, unlike ß cells, suppression of apoptosis through mutation is not required for the establishment and progression of superficial skin tumours. However, it is of note that c-Myc-induced papillomas remain completely non-invasive yet in the absence of p53 they rapidly invade the underlying dermis and mesenchyme. As p53 is required for efficient c-Myc-induced apoptosis in keratinocytes, the primary role for p53 loss may be to allow survival of invading keratinocytes when migrating into an ‘inappropriate’ trophic environment. These two transgenic models exemplify how different ‘rules’ govern tumorigenesis and progression in tissues reflecting differing architectures and functions and may shed light on why neoplasms in differing locations adopt preferred evolutionary routes to malignancy.

### S9 MODELLING INTESTINAL NEOPLASIA

W Dove, A Bilger, R Cormier, K Gould, K Haigis, R Halberg, A Lillich, A Merritt, A Shedlovsky, A Shoemaker and A Thliveris, McArdle Laboratory and Laboratory of Genetics, University of Wisconsin, Madison, WI 53706 USA

The Min mouse strain, predisposed to multiple intestinal polyposis, is heterozygous for an ENU-induced nonsense allele of *Apc*, the mouse homolog of the human gene *Adenomatus polyposis coli*. The initial phase of studies with *Min* and other targeted alleles of *Apc*, worldwide, has generated both advances and *challenges* in reaching a deeper understanding of human colon cancer.

- The multiplicity of adenomas in mice carrying the *Min* allele is strongly affected by the genetic background, varying over two orders of magnitude. Studies in different laboratories can be compared more rigorously when both the *Apc* allele and the genetic background are standardized. *Can mouse models with validated genetic constitution be effectively distributed worldwide*?
- Adenoma formation commonly involves loss of expression of the wildtype Apc allele, but not always through allele loss or intragenic mutation. *What Apc-silencing mechanisms exist?*
- Adenomas are commonly polyclonal in structure, with each contributing line-age showing *Apc* loss. *Is polyclonality passive or does it involve active cooperation in the transition to or maintenance of neoplasia? What is the primary rate of allele-loss at the Apc locus*?
- The maintenance DNA methylase positively regulates the net growth rate of adenomas. *What are the salient growth-regulatory genes through which DNA methylation operates?*
- The *p53* gene modestly affects adenoma multiplicity, but *p53* mutations lead to a small set of locally invasive intestinal tumors in Min mice. *Can invasive and metastatic intestinal tumors be made a more prominent component of the tumor spectrum in Min and other mutant mice?*
- The multiplicity and net growth rate of adenomas is affected by polymorphic modifier loci. The first such locus to be investigated in great detail, *Mom l*, involves a secretory phospholipase synthesized by Paneth cells and goblet cells in resistant strains. However, there is at least one other gene that accounts for the resistance phenotype in the *Mom l* region. *Can the complexity of modifier loci be reduced, for example by point mutagenesis of the germline? Does the secretory phospholipase affect colonic adenomas in the human?*
- One positive cellular marker has been found for the proliferative compartment within intestinal crypts and intestinal tumors in the mouse: *ROSA*11. *Can diagnostic cellular markers be developed to discriminate among intestinal tumors of differing biological source and potential?*

Dove WF, Cormier RT, Gould KA, Halberg RB, Merritt AJ, Newton MA and Shoemaker AR (1998) *Phil Trans R Soc, Lond B*, **353:** 915.

### S10 MODELING CANCER IN THE MOUSE

Tyler Jacks, Howard Hughes Medical Institute, Center for Cancer Research, Department of Biology, Massachusetts Institute of Technology, Cambridge, MA 02139 USA

The advent of gene targeting technology in mouse embryonic stem (ES) cells has made it possible to introduce specific mutations into the murine germline. Over the past several years, we have used this technology to mutate the murine homologs of a series of human genes implicated in tumor development, specifically the tumor suppressor genes Rb, p53, Nf1 and Nf2. These mutant animals have been useful as models for certain human familial cancer syndromes caused by the inheritance of a single mutant allele of a given tumor suppressor gene, as a means to address the role for these genes in normal development, and as a source of primary cells and cell lines with which to examine tumor suppressor gene function in vitro. Recently, we have also developed a novel mouse strain carrying a targeted mutation in the K-ras proto-oncogene, which causes predisposition to tumors of the lung and other sites. These mutant strains will be described with an emphasis on our attempts to address species-specific differences in the response to inherited mutations in cancer-associated genes as well as our efforts to construct more accurate murine models of human cancer, both genetically and histopathologically.

### S11 NEW MOUSE MODELS FOR HUMAN CANCER

Anton Berns, Division of Molecular Genetics and Centre of Biomedical Genetics, The Netherlands Cancer Institute, Plesmanlaan 121, 1066 CX, Amsterdam, The Netherlands. e-mail: tberns@nki.nl

Gene inactivation studies are invaluable in assessing the function of oncogenes and tumor suppressor genes in development and malignant growth. However, detailed analysis of the role of tumor suppressor genes in these processes using the conventional knockout mouse models is often hampered by embryonic lethality or developmental aberrations.

To circumvent these complicating factors associated with loss-of-tumor suppressor gene function we have generated a series of conditional tumor suppressor gene knockout mice using the Cre/Lox system. We have explored methods to switch these genes off in a time-controlled and tissue specific fashion. Both transgenesis and somatic gene transfer was used to express Cre recombinase in the desired tissues. This technology permits us to induce specific tumors, and to correlate specific genetic lesions with phenotypic characteristics. Since we now can induce multiple defined mutations within the same cell of a tissue we can mimic closely the genetic aberrations found in tumors in man. This will facilitate the testing of intervention strategies targeted to distinct pathways disrupted in the model in conjunction with a range of other mutations. To follow tumor growth in vivo we have implemented new imaging techniques.

These compound mutant mice are also a valuable source of cell lines that can be tested with respect to parameters that are better studied in vitro such as growth, cell cycle regulation, response to irradiation, resistance to apoptosis, and genomic instability. Examples will be discussed.

### S12 INVESTIGATION INTO THE PHARMACODYNAMICS OF THE BROAD-SPECTRUM NEUROPEPTIDE GROWTH FACTOR ANTAGONISTS IN THE EARLY CLINICAL DEVELOPMENT OF ANTAGONIST G

S.Clive, ICRF Medical Oncology Unit, Western General Hospital, Crewe Road, Edinburgh, EH4 2XU.

Small cell lung cancer (SCLC) cells secrete neuropeptides that act as autocrine growth factors. Arg-D-Trp-NmePhe-D-Trp-Leu-Met-NH2 (Ant G), a substance P (6-11) analogue, antagonises a broad spectrum of neuropeptides, including vasopressin (AVP), gastrin releasing peptide (GRP) and bradykinin (BK), has *in vivo* activity in SCLC models and was selected for clinical development. A phase I trial was performed, under the auspices of the CRC Phase I/II Committee (CRC 90-08), in 2 stages. The primary aim of stage I was to achieve an end of infusion Ant G plasma concentration ($C_{max}$) of 10µM, a concentration associated with anti-tumour activity pre-clinically. Given by 6-hour, 3 weekly infusion through a central line, dose was escalated from 2–300 mg/m$^2$ with 15 patients entered (12M: 3F; age 36–65 (median 59), PS median 1). $C_{max}$ of 10µM was exceeded in 3/3 patients at 300mg/m$^2$ (13–24 µM). In stage 2, dose intensity was increased (weekly infusion) and forearm blood flow (FBF) changes as measured by venous occlusion plethysmography were incorporated as a pharmacodynamic endpoint. 9 patients (5M: 4F; age 34–75 (median 54), PS median 1) were entered in 3 dose levels, achieving $C_{max}$ 19–45(M at the highest (400mg/m$^2$) dose. Toxicity related to facial flushing and was not dose limiting. FBF studies were performed in 8/9 patients. Intra-arterial infusions of neuropeptides, substance P (SP) and bradykinin (BK), were performed before and during Ant G infusion. Ant G caused a significant ($p<0.05$) reduction in the arterial dilatory effects of SP (66%) and BK (33%). Although the validity of these studies as surrogate markers of activity of Ant G requires confirmation, biological activity of Ant G was demonstrated in patients at $C_{max}$ at which preclinical antitumour activity had been seen. This was accepted for completion of the phase I trial without definition of the MTD and a dose of 400 mg/m$^2$ has been suggested for phase II studies. The mitogenic properties of SP in SCLC remain undefined whereas GRP and AVP are recognised autocrine growth factors in SCLC and Ant G is a potent antagonist of both *in vitro*. Therefore the effects of GRP and AVP on FBF in healthy volunteers was examined. GRP caused consistent, dose-dependent vasodilatation at doses that could be given intra-arterially without toxicity, a finding that has not previously been demonstrated. Therefore it would be a suitable neuropeptide for use in FBF studies in patients receiving Ant G. AVP did not demonstrate a consistent response in FBF studies. However, AVP is known to cause aggregation of platelets in platelet rich plasma (PRP) and the effects of Ant G on this were examined in volunteer PRP. Ant G was consistently and selectively shown to inhibit AVP-induced platelet aggregation in a competitive, dose-dependent manner. This assay is relatively non-invasive, would enable a variety of time-points to be examined and would be attractive as a pharmacodynamic measure for inclusion in the further clinical development of Antagonist G.

### S13 REGULATION OF ETS TRANSCRIPTION FACTOR ACTIVITY

Shen-Hsi Yang[1], Alex Galanis[1], Elaine Vickers[1], Amanda Greenall[2], Paula Yates[2], Nicola Willingham[2] and *Andy Sharrocks*[1], [1]School of Biological Sciences, University of Manchester, Manchester, UK. [2]School of Biochemistry and Genetics, The Medical School, University of Newcastle Upon Tyne, Newcastle Upon Tyne, UK

Several members of the ETS-domain transcription factor family have been implicated in tumorigenesis. For example, the founder member Ets-1 is a proto-oncogene. Further ETS-domain proteins are deregulated by either overexpression or rearrangement of their genes, resulting in a variety of different types of cancers. In the majority of these tumours, the changes seen in individual proteins, generally result in retention of their intact DNA binding domains but either deletion or alterations in their regulatory regions.

The ETS-domain transcription factors are often regulated by MAP kinase pathways, components of which are targets for deregulation during tumour formation. In recent years, our understanding of the molecular function of different ETS-domain proteins has increased substantially. Our recent studies emphasise the importance of the ETS-domain and the regulatory regions in their function and hence extend our understanding of the molecular causes of tumorigenesis.

The ETS-domain transcription factor Elk-1 and the related proteins SAP-1 and SAP-2 play a pivotal role in the regulation of c-*fos* in response to extracellular signals. We have demonstrated that divergent MAP kinase pathways transduce these signals and their specificity of action involves the complex interplay of a series of docking/targeting motifs. Phosphorylation of the transcription factors results in their activation. One mechanism by which this occurs is by changing the conformation of the proteins, which potentially uncovers/masks interaction surfaces for coregulatory proteins. Recent data indicate that this is indeed a distinct possibility and coactivator/corepressor complexes can be recruited following phosphorylation of transcription factors by MAP kinases. Further complexities in the activation/repression mechanisms have also been revealed. Finally, novel links have been uncovered between the Id helix-loop-helix proteins and ETS transcription factors where the Id proteins downregulate the activity of ETS-domain transcription factors.

Collectively, our data enhance our knowledge of how this important class of transcription factors functions at the molecular level, how they interact with the MAP kinase pathways and how the activities of ETS-domain proteins might be disrupted during tumorigenesis.

### S14 TECHNOLOGICAL DEVELOPMENTS IN EXTERNAL BEAM RADIOTHERAPY

PC Williams* and JM Wilkinson, North Western Medical Physics, Christie Hospital, Manchester M20 4BX, UK

The technology for radiotherapy has developed continually since the introduction of the linear accelerator. Certain landmark developments include the introduction of computerised treatment planning based on CT imaging and the introduction of multileaf collimation facilitating practical application of 3D conformal therapy.

Conformal radiotherapy is not a well defined term but includes any treatment technique which intends to make the treated volume closely match the target volume has been termed conformal radiotherapy. The purposes of conformal therapy are either to reduce the target volume and hence allow the possibility of dose escalation or to minimising the complication rate by reducing the volume of healthy and susceptible tissue included in the treated volume.

Conformal therapy demands high precision in the localisation, planning and delivery of treatment which then needs to be verified to ensure that the geometric and dosimetric requirements of each treatment are achieved. Electronic portal imaging has addressed this requirement.

Standard conformal therapy is now widely, but not universally, available. Studies have shown that significant benefits can be realised by such techniques which exploit the ease at which treatment beams can be shaped, by an MLC, to match the projects of targets to be irradiated and sensitive structures to be shielded.

Static use of an MLC generates 3 dimensional target volumes by the intersection of beams shaped in 2 dimensions and projected from different directions. It does not address the problem of generating non uniform intensities to compensate for the varying attenuation and scattering in tissues through which the radiation must pass, nor does it address the problem of shaping the treatment volume in the third dimension as is required if the target volume is invaginated in the planes normal to the rotation axis of the accelerator.

Both these problems can be addressed by the use of Intensity Modulated Radiotherapy (IMRT) in which the positions of the Multileaf collimator are varied during irradiation. The current state of development of IMRT will be outlined with particular reference to the clinical applications that have been implemented.

Following the full implementation of IMRT. Our ability to deliver accurately highly modulated beams will exceed our ability to define the target tissues and to verify their position during irradiation. Further development of Image Guided Radiotherapy will then be justified.

### S15 CLINICAL IMPLEMENTATION OF INTENSITY MODULATED RADIOTHERAPY

W De Neve, Division of Radiotherapy, Ghent University Hospital, B-9000 Ghent, Belgium

Intensity modulated radiotherapy (IMRT) is the hot topic in radiotherapy translational research. IMRT allows us to customize the dose distribution to maximize the probability of uncomplicated local control. Our initial focus for clinical implementation was on head and neck and prostate cancer.

**Methods** IMRT planning consisted of a mixture of forward (class solution)[2] and inverse [desired dose distribution decomposition; intensity optimization elements (W. De Neve, Presidential Course, ASTRO 1999)][3,1,2]. At the end stage of planning, segments were linked for fast sequential delivery by an SL-18-MLCi or SL-25-MLCi (Elekta), with a dynamic multileaf collimator forced in step and shoot mode[2].

**Results** Between 1/9/1996 and 31/10/1999, 32 patients with cancer in the head and neck region were treated using segmental IMRT. Eleven of these patients were re-irradiated for inoperable relapses or second primaries in previously irradiated regions. All re-irradiated patients had severe symptoms. For 8 of 10 evaluable patients, palliation was achieved. Median duration of response was 9 months. Median survival calculated from the onset of re-irradiation was 15 months. The cause of death was intercurrent (intestinal hemorragh) in one patient and related to disease progression in five patients. Subcutaneous fibrosis was reported in seven patients; temporomandibular joint impairment and laryngeal edema each in one patient. In spite of cumulative doses as high as 136 Gy, no cases of cranial nerve palsy, arterial rupture or bone necrosis were observed. From July 1998 till December 1999, 31 patients received IMRT for prostate cancer to a prescription median dose of 76 Gy in 36 fractions. The main goal of IMRT was to keep the maximal rectal dose at 72 Gy. No acute toxicity above grade 2 was observed. For head and neck tumours, the delivery of 20–50 segments per session took 12–22 minutes, including the time required for patient setup and portal imaging. Prostate IMRT (13–20 segments) was delivered in 6–12 minutes depending on the ease of patient setup and on portal imaging.

**Discussion** During recent years, MLC technology capable of delivering multisegment or dynamic IMRT became available. Like all other centers that have applied IMRT with large MLCs (i.e. not NOMOS MiMiC) during the last decade, we had to solve the IMRT planning problem in-house which involved the development, interfacing maintenance and quality assurance of IMRT planning tools. Actually, about 10% of our patients receive IMRT making the projected total number of IMRT treatments about 100 for the year 2000. With the fast progress in the field, we feel the need to participate in inter-institutional, preferably randomized IMRT studies.

1 W De Gersem et al (1999) *Int J Radiat Oncol Biol Phys* **44:** 461–468
2 W De Neve et al (1999) *Radiother Oncol* **50:** 301–314
3 C. De Wagter et al (1998) *Radiother Oncol* **47:** 69–76

### S16 BRACHYTHERAPY FOR LOCALISED PROSTATE CANCER

Dr D Ash, Cookridge Hospital, Leeds, UK

As a result of PSA testing an increasing number of patients with localised prostate cancer are presenting for treatment; many are less than 70 years old and suitable for radical local therapy. The conventional alternatives are external beam radiotherapy or radical prostatectomy. New techniques of brachytherapy now offer a much less invasive alternative with a lower risk of side effects.

The majority of patients suitable for radical prostatectomy are also suitable for brachytherapy. The selection factors that determine probability of PSA control are the same ie presenting PSA, Gleason grade and stage.

The factors which determine functional outcome are different. For prostatectomy, age and co-morbidity are important whereas it is less of a problem for brachytherapy. For brachytherapy significant outflow obstruction may increase the risk of obstruction and delayed resolution of urethral side effects. For both treatments the quality of the intervention has a significant bearing on outcome. For surgery the margin status is highly significant. For brachytherapy the dosimetric quality which confirms that the tumour has been enclosed within the treatment isodose is also highly significant. When comparing treatments it is therefore necessary to know not only what the pre-treatment characteristics are but also the quality of the intervention.

Selected patients treated by high quality dosimetry controlled brachytherapy often leave hospital the same or the next day after the implant and many are back at work within seven days. The risk of incontinence is less than 1% and the risk of impotence approximately 30%. At ten years approximately 80% of good prognosis patients remain biochemically controlled. Because of the convenience, low risk profile and equivalent outcome brachytherapy is preferable to radical prostatectomy for a high proportion of patients.

### S17 CHEMO-RADIOTHERAPY: IN PURSUIT OF THE MYTHICAL FREE LUNCH

Alastair J Munro, Dept of Radiotherapy, Ninewells Hospital and Medical School, Dundee DD1 9SY, UK

A succession of clinical trials comparing radiotherapy alone with radiotherapy plus synchronous chemotherapy have suggested that combined treatment improves both local control and survival. These results suggest, at face value, that there may be a truly supra-additive interaction between drugs and radiation. More detailed analysis of the data is less convincing. In trials of chemo-radiotherapy in cancers of the head and neck the improvement in local control and survival is paralleled by an equivalent increase in acute toxicity. The results from the recent, much publicised, studies in cancer of the cervix are similar. In randomised studies of chemo-radiotherapy in cancers of the anus and rectum there is, again, a higher rate of acute toxicity with combined treatment. There is also a disconcerting increase in non-cancer deaths, suggesting that late effects of treatment may contribute to an increase in mortality with combined treatment. The overall conclusion is that, before wholeheartedly embracing combined chemotherapy and radiation, we need to have better data on acute toxicity, on late effects and on patients' views concerning any trade-offs between the benefits and harms associated with combined treatment. For the moment at least, we are left wondering whether the apparent benefits achieved using combined treatment could have been achieved with radiation alone, simply by increasing the dose.

### S18 INTEGRATING THE CELL DEATH PATHWAY

Stanley J Korsmeyer, Dana-Farber Cancer Institute, One Jimmy Fund Way, Rm SM758, Boston, Massachusetts 02115, USA

BCL-2 has the novel function of blocking the programmed death of cells. BCL-2 protein duels with its counteracting partner known as BAX. An expanding family of BCL-2 related proteins shares homology clustered within four conserved regions called BCL-2 homology (BH) 1 through 4. These novel domains control the ability of these proteins to dimerize and function. Following a death stimulus, the monomeric, cytosolic BAX translocates to mitochondria where it is a homodimerized, integral membrane protein that activates apoptosis. BAD resides more proximal in the pathway and determines the effectiveness of BCL-2 or BCL-$X_L$. In the presence of survival factor IL-3, cells phosphorylated BAD on two serine residues embedded in 14–3–3 consensus-binding sites. This frees BCL-$X_L$ to repress cell death. The rapid phosphorylation of BAD connects a proximal survival signal with the BCL-2 family, utilizing distinct kinases to inactivate BAD by selective phosphorylation of Ser112 or Ser136. PKA, which is tethered to mitochondria by an A-Kinase Anchoring Protein (AKAP), is responsible for the BAD Ser112 phosphorylation, representing a focused interaction that inactivates BAD at its target organelle. BID possesses sequence homology only at the BH3 domain and is found in both cytosolic and membrane locations. A multidimensional NMR structure indicates p22 BID has a similar α helical structure to BCL-$X_L$ and undergoes structural changes upon activation. An intact BH3 domain of BID is required for it to bind BCL-2 or BAX as well as to promote apoptosis BH3-only molecules support BH3 as the minimal death domain and are candidates to connect with proximal signal transduction. TNFα and FasL induce a Caspase-8-mediated cleavage of the cytosolic, inactive p22 BID to create a predominant p15 BID that translocates to mitochondria as an integral membrane protein. P15 BID appears to be required for the release of cytochrome c. *Bid*-deficient mice proved resistant to Fas-induced hepatic apoptosis, indicating it is a singularly important Caspase substrate. BCL-2 family serves as a checkpoint in the cell death pathway in which activation of pro-apoptotic members such as BAX, BAD or BID result in a conformational change, a translocation from cytosol to mitchondria and the initiation of an apoptotic program including Caspase activation and mitochondrial dysfunction.

### S19 MOLECULAR REMISSION IN FOLLICULAR LYMPHOMA THEORETICAL CONCEPT OR THERAPEUTIC GOAL?

Dr A Rohatiner, Dept of Medical Oncology, St. Bartholomew's Hospital, London EC1A 7BE, UK

With conventional therapy, follicular lymphoma remains demonstrably incurable for most patients; an experimental approach is therefore justified. Recognition of the association between follicular lymphoma and the t(14;18) translocation and the possibility of detecting residual disease at the molecular level using PCR analysis, has led to the concept of 'molecular remission'. If the presence of PCR-detectable, residual t(14;18) containing cells is used as a surrogate marker for the disease activity, PCR analysis can be used to access the efficacy of a treatment. The hypothesis therefore, (to be confirmed or refuted), is that achievement of response at the 'molecular level' has prognostic implications and is therefore a worthwhile aim.

Several new approaches, including the chimeric antibody anti-CD20, radiolabelled murine anti-CD20 and the combination of Fludarabine, Mitoxantrone and Dexamethasone have all been reported to result in 'molecular remission' in a proportion of patients. The significance of these findings is currently unknown. High dose treatment (Cyclophosphamide and total body irradiation) supported by autologous haemopoietic progenitor cells has also led to 'molecular remissions' in some patients, with apparent prognostic significance. The use of 'real-time' PCR in the future may help to clarify the situation.

Data relating to these concepts and treatments will be discussed.

### S20 HEPATITIS B (HBV) INFECTION, LYMPHOMA AND BONE MARROW TRANSPLANTATION (BMT)

Raymond Liang MD, Division of Haematology and Oncology, Department of Medicine, University of Hong Kong, Queen Mary Hospital, Hong Kong

HBV infection is endemic in many parts of Asia and Africa. In many of these places, a chronic HBV carrier rate of over 10% is observed. The infection is predominantly transmitted perinatally from the mother to the baby. Early contact to the virus probably has a higher chance of resulting in a chronic carrier state.

In Hong Kong, 10% of the population is serum HBsAg positive and another 40% with the protective antibody HBsAb positive, indicating that half of the population has had prior exposure to the virus. A similar pattern is observed in cancer patients except lymphoma. At the time of diagnosis, at least 20% of the lymphoma patients in Hong Kong are HBsAg positive and only 30% HBsAb positive. This may be explained by the immunosuppressive effect of the lymphoma, resulting in about 10% of the originally HBsAb positive patients turn HBsAg positive. There is currently no evidence to suggest that HBV infection is aetiologically related to lymphoma.

Reactivation of HBV infection is common after chemotherapy for lymphoma. As high as 50% of the HBV carriers may develop clinical hepatitis, 10% liver failure. The liver related mortality is about 5%. The immunosuppressive effect of the chemotherapy results in increased viral replication in the liver. Upon cessation of the chemotherapy and recovery of immunity, there is a marked immune mediated destruction of the heavily virus-loaded liver cells. A surge in serum HBV DNA level after chemotherapy seems to be a good early marker of HBV reactivation. The new antiviral agents active against HBV, such as lamivudine, are very effective in preventing this potentially fatal complication. Screening of HBV markers for all lymphoma patients from endemic areas is an essential part of the protocol.

The problem is more serious if chronic HBV carriers are required to receive autologous or allogeneic stem cell or bone marrow transplantation. There is a high liver related morbidity or mortality. Anti-viral therapy has also been shown to play an important role in this setting. On the other hand, serological clearance of HBV has been observed commonly after BMT in HBsAg positive recipients using HBsAb positive marrow. Transfer of humoral and cellular immunity to the patient from donor has resulted in seroconversion from HBsAg to HBsAb positivity.

### S21 EPSTEIN-BARR VIRUS POSITIVE LYMPHOMAS: PROSPECTS FOR IMMUNOTHERAPY

Alan Rickinson, CRC Institute for Cancer Studies, University of Birmingham, UK

### S22 WHY DO WE DO WHAT WE DO? A LOOK AT THE RATIONALE, INDICATIONS AND PERSPECTIVES OF RADIOTHERAPY

Jens Overgaard, Danish Cancer Society, Department of Experimental Clinical Oncology, Aarhus University Hospital, Aarhus C, Denmark

Despite significant focus on education, evidence-based medicine, and other attempts to develop rational therapeutic strategies in cancer treatment, the practice of radiotherapy widely differs among departments and national policies. With this in mind, the indications, rationale, and strategies for optimal radiotherapy will be discussed in the light of recent development in technical and biological skills.

Radiotherapy is, next to surgery, the most important curative modality in cancer treatment. Thus more than 80 percent of cancer cures are obtained with these two loco-regional modalities, either alone or combined. At present approx. half of all cancer patients will receive radiotherapy, either as a part of their primary treatment and/or in palliative settings. The role of radiotherapy is increasing due to new indications and attempt to conserve organs and functions. This can be seen in new therapeutic strategies of e.g. early breast cancer, head and neck carcinoma, colo-rectal carcinoma and prostate cancer, and importantly, palliation. The development in radiotherapy has throughout the century been alternating between technical improvement and biological optimization. Thus the last decade did especially result in an improved technical ability to focus high dose delivery of accurate radiation, especially due to improvement in computer and imaging technology. The upcoming years will in contrast be expected to utilize the new molecular biological knowledge in an attempt to understand and take advantage of the fundamental mechanisms of the radiation effects. This will among others be directed towards reducing the dose-limiting morbidity.

Thus, the focus on cancer in the start of the new millennium will be maintained on the current loco-regional treatment modalities, but under influence of recent and expected achievements in technical and biological knowledge. Utilization of such improvements will request a responsive community of well, and continuously, educated cancer specialists working in a multi-disciplinary and multi-institutional team work.

### S23 ROLE OF TGFβ SIGNALING IN SKIN CARCINOGENESIS

Cindy Go[1], Brian Weeks[1], Wei He[2], Kristin M Liefer[3], Tongyu Cao[3], and Xiao-Jing Wang[2,3] Department of [1]Otolaryngology, [2]Dermatology, [3]Molecular and Cellular Biology, Baylor College of Medicine, Houston, TX 77030, USA

The TGFβ signaling pathway is one of the most important mechanisms in the maintenance of epithelial homeostasis. Alterations leading to either the repression or enhancement of this pathway affect cancer development. We have examined expression of TGFβ signaling components during skin chemical carcinogenesis, and found that although TGFβ1 was overexpressed, TGFβ receptors or signaling Smads were lost. To further understand the pathological consequence of these changes, we have generated complimentary transgenic mouse models, which express either a dominant negative TGFβ type II receptor (ΔβRII) or TGFβ1 in the epidermis. The ΔβRII mice exhibited an increased susceptibility to a chemical carcinogenesis protocol, with increased tumor formation and early metastasis. ΔβRII tumor cells showed accelerated aberrant cell cycle progression compared to tumors developing in non-transgenic mice. Consistently, ΔβRII tumors showed reduced expression of p15, p21 and p27, inhibitors of cyclin-dependent kinases (cdks). Although gDβRII tumors exhibited early metastasis, most ΔβRII carcinomas did not exhibit chromosome instability, but exhibited increased angiogenesis and matrix metalloproteinases, which correlated with overexpression of the endogenous TGFβ1. These data suggest that tumors which possess both elevated TGFβ and loss of functional TGFβ receptor may have poor prognosis, i.e., the mutant TGFβ receptor allows tumor cells to escape from TGFβ-induced growth arrest, whereas elevated endogenous TGFβ facilitates tumor invasion via a paracrine effect. This hypothesis is now being further tested by utilizing the gene-switch-TGFβ1 mice, which allow the induction of TGFβ1 transgene expression at different stages of skin carcinogenesis.

1 Go C, Li, P, Wang XJ (1999). Blocking TGFβ signalling in transgenic epidermis accelerates chemical carcinogenesis via mechanisms associated with aberrant cell cycle progression and angiogenesis. *Cancer Res* **59:** 2861–2868

2 Wang XJ, Liefer KM, Tsai S, O'Malley BW, Roop DR (1999) Development of Gene-Switch mice which inducibly express transforming growth factor β1 (TGFβ1) in the epidermis. *Proc Natl Acad Sci, USA* **96:** 8483–8488

### S24 KERATIN K10, FROM CELL STRUCTURE TO CELL CYCLE THROUGH SIGNAL TRANSDUCTION

JM Paramio, C Segrelles, MIL Casanova, M Santos, S Ruiz and JL Jorcano Dept of Cell and Molecular Biology. CIEMAT. Av. Complutense 22, E-28040 Madrid, Spain

The members of the large keratin family of cytoskeletal proteins are expressed in a precisely regulated tissue- and differentiation stage-specific manner. Although these proteins are thought to be involved in conferring mechanical integrity to epithelial cells, the functional significance of their complex differential expression is still unclear. In epidermis, basal proliferative keratinocytes express K14; when they terminally differentiate, keratinocytes switch off K14 and start K10 expression, whereas in response to hyperproliferative stimuli, K16 replaces K10.

We have recently demonstrated that the ectopic expression of K10 inhibits proliferation of human keratinocytes in culture, whilst K16 expression appears to promote proliferation of these cells. In addition, K10-induced inhibition can be reversed by co-expression of K16, but not K14. The mechanism by which K10 inhibits cell proliferation is linked to the retinoblastoma pathway. We found that this effect on cell cycle progression can be attributed to alterations in the PI-3 kinase signal transduction pathway mediated by the physical interaction between the non α-helical aminoterminal domain of K10 and Akt/PKB and aPKCζ. This interaction prevents the translocation of these signalling molecules, thus impairing their activation. Finally, to confirm these data *in vivo*, we generated transgenic mice in which K10 expression was ectopically targeted to the basal layer of the epidermis. These mice showed striking alterations in epidermal proliferation and differentiation, and displayed a highly decreased sensitivity to skin chemical carcinogenesis. The biochemical analysis of this phenotype was in agreement with the *in vitro* results.

### S25 GENETIC MODELS FOR SKIN CANCER Petra Boukamp, DKFZ, Heidelberg, Germany

Despite an increasing number and agressiveness of non-melanoma skin cancer relatively little is known about specific genetic aberrations and their underlying mechanisms. We have developed an in vitro model of skin carcinogenesis starting with the spontaneously immortalized human HaCaT skin keratinocytes. In this model, mutational inactivation of p53, aneuploidy and particularly loss of chromosome 3p (introduction of a normal copy of chromosome 3 can revert immortality), as well as telomerase upregulation were early events, correlating with immortalization. Tumor progression could be induced by introduction of the Harvey-ras oncogene as well as by long-term treatment of these cells with increased temperature. This latter treatment caused DNA strand breaks which resulted in genomic instability and a tumorigenic phenotype which was correlated with gain of chromosomal material of 11q13 and overexpression of cyclin D1, mapped to 11q13. In both cases, the tumor phenotype depended on the recipient cell. Early passage HaCaT cells developed benign tumors and late passage cells malignant tumors when injected s.c. into nude mice suggesting that the Ha-ras gene as well as the 11q13 amplification were responsible for unrestricted benign-tumorigenic growth but not malignancy. Malignant conversion, on the other hand, could be related to loss of a copy of chromosome 15. By using a skin squamous cell carcinome cell line, SCL-I we were able to demonstrate that loss of chromosome 15 was causal for tumorigenic progression by reducing the level of thrombospondin 1, mapped to 15q15, and by allowing tumor vascularization and invasive growth. Thus, loss of chromosome 15 was responsible for the angiogenic switch during tumor progression.

In agreement, an in vivo skin progression model (MET lines derived from a primary-, recurrent- and metastatic lesion) showed aneuploidy, loss of 3p, and gain of 11q13. Polyploidization, common in cultured cell lines, was only seen in the cell line established from the metastasis indicating that polyploidization may be a late event in skin carcinogenesis in man. Interestingly, all MET cells were wildtype for p53 (the first SCC lines) and all ras genes and were deficient of HPV thus, making them the first model to study p53 alternative pathways in skin cancer.

### S26 THE SEARCH FOR EPIDERMAL STEM CELLS Fiona M Watt, Imperial Cancer Research Fund, London WC2A 3PX, UK

The epidermis is renewed throughout adult life through proliferation of a subpopulation of cells in the basal layer, known as stem cells. Stem cells have the capacity to self-renew and also to generate daughter cells that undergo terminal differentiation. Human epidermal stem cells can be maintained in culture and this makes it possible to evaluate potential markers of the stem cell compartment and to identify factors that regulate stem cell fate. I shall describe recent progress in identifying stem cell markers and in using these to reveal the highly patterned distribution of the cells in normal human epidermis. I shall also describe factors that act cell-autonomously to regulate stem cell self-renewal and factors that mediate interactions between stem cells and other basal layer cells. Finally I shall compare human epidermal stem cells in vitro with stem cells in vivo, in transgenic mouse epidermis.

### S27 ANALYSIS OF TUMOUR SUSCEPTIBILITY USING MOUSE MODELS OF HUMAN CANCER Allan Balmain, Rosemary Akhurst, Martin Oft, Hiroki Nagase, Jian Hua Mao

Much of our knowledge of the processes of multistage carcinogenesis has come from studies of rodent models in which both the genetic background of the host and the etiology of tumor development can be carefully controlled. Studies of skin tumorgenesis have identified many of the somatic genetic changes in tumor initiation and progression, the biological events associated with these genetic alterations, and the role of host genetic background in determining tumor susceptibility. Genetic changes in skin carcinogenesis in the mouse resemble those seen in human squamous carcinomas, and include point mutations in ras and p53, and deletions at the p16INK4 locus. The rodent models have allowed us to determine the stages at which these changes occur, and to demonstrate the importance of the target cell (stem cell or committed cell) in which mutations take place. Quantitative changes in signal strength through the ras pathway are also important as tumors progress to malignancy.

Studies of cell lines derived from different stages of carcinogenesis have allowed us to identify co-operative interactions between ras and TGFβ signalling pathways in the control of tumor metastasis. Finally, the use of classical mouse genetic approaches has led to the identification of multiple loci in the mouse genome that are polymorphic between different mouse strains, and have major effects on germline susceptibility to tumors induced by exposure to carcinogens. These mouse models provide us with a novel route to the identification of tumor modifier genes that have implications for future approaches to prevention, diagnosis and therapy of human cancers.

